# Congenital brain abnormalities during a Zika virus epidemic in Salvador, Brazil, April 2015 to July 2016

**DOI:** 10.2807/1560-7917.ES.2018.23.45.1700757

**Published:** 2018-11-08

**Authors:** Mariana Kikuti, Cristiane W. Cardoso, Ana P.B. Prates, Igor A.D. Paploski, Uriel Kitron, Mitermayer G. Reis, Ganeshwaran H. Mochida, Guilherme S. Ribeiro

**Affiliations:** 1 Fundação Oswaldo Cruz, Salvador, Brazil; 2 Universidade Federal da Bahia, Salvador, Brazil; 3 Secretaria Municipal de Saúde de Salvador, Salvador, Brazil; 4 Emory University, Atlanta, United States; 5 Boston Children’s Hospital, Boston, United States; 6 Massachusetts General Hospital, Boston, United States; 7 Harvard Medical School, Boston, United States

**Keywords:** microcephaly, brain abnormalities, Zika virus, prevalence, sensitivity and specificity, nervous system malformation

## Abstract

**Background:**

North-eastern Brazil was the region most affected by the outbreak of congenital Zika syndrome that followed the 2015 Zika virus (ZIKV) epidemics, with thousands of suspected microcephaly cases reported to the health authorities, mostly between late 2015 and early 2016. **Aim:** To describe clinical and epidemiological aspects of the outbreak of congenital brain abnormalities (CBAs) and to evaluate the accuracy of different head circumference screening criteria in predicting CBAs.

**Method:**

Between April 2015 and July 2016, the Centers for Information and Epidemiologic Surveillance of Salvador, Brazil investigated the reported cases suspected of microcephaly and, based on intracranial imaging studies, confirmed or excluded a diagnosis of CBA. Sensitivity, specificity and positive and negative predictive values of different head circumference screening criteria in predicting CBAs were calculated.

**Results:**

Of the 365 investigated cases, 166 (45.5%) had confirmed CBAs. The most common findings were intracranial calcifications and ventriculomegaly in 143 (86.1%) and 111 (66.9%) of the 166 CBA cases, respectively. Prevalence of CBAs peaked in December 2015 (2.24 cases/100 live births). Cases of CBAs were significantly more likely to have been born preterm and to mothers who had clinical manifestations of arboviral infection during pregnancy. None of the head circumference screening criteria performed optimally in predicting CBAs.

**Conclusion:**

This study highlights the magnitude of neurological consequences of the ZIKV epidemic and the limitations of head circumference in accurately identifying children with CBA. Gestational symptoms compatible with ZIKV infection should be combined with imaging studies for efficient detection of suspect CBAs during ZIKV epidemics.

## Introduction

Early in 2015, large outbreaks of acute exanthematous illness were detected in several states in north-eastern Brazil. By April 2015, Zika virus (ZIKV) was identified as the aetiology of the illness [1,2]. A few months after the epidemic peak in May 2015, an increase in newborns with microcephaly was noted in north-eastern Brazil [3] and promptly gathered global attention due to a possible link between gestational ZIKV infection and microcephaly. Since then, evidence for a causal association between in utero exposure to ZIKV and microcephaly and other neurological complications has emerged [4-6]. The constellation of clinical manifestations of congenital ZIKV infection may be referred to as ‘congenital Zika syndrome’ [7].

In light of the surge of microcephaly cases, the Brazilian Ministry of Health (BMoH) declared a national public health emergency in November 2015 and initiated a surveillance programme for identification of suspected microcephaly cases [8]. All health facilities were required to report such cases [8-10] and encouraged to report spontaneous abortions and stillbirths in women with a history of a rash during pregnancy, in a national reporting system. Although head circumference was immediately adopted as the primary criterion for screening cases suspected of congenital abnormalities by Zika, not all children with neurological impairment due to ZIKV present with microcephaly at birth [11]. Therefore, it is important to understand how well the criteria used to detect microcephaly can predict the congenital brain alterations of ZIKV.

Here, we describe the characteristics of the cases with congenital brain abnormalities (CBAs) confirmed by intracranial imaging studies among the reported cases of suspected microcephaly in Salvador, Brazil. We also identified clinical manifestations during pregnancy that were associated with CBAs, and evaluated the accuracy of different screening criteria based on head circumference for predicting CBAs.

## Methods

### Suspected microcephaly case definition for mandatory reporting

From 17 November to 11 December 2015, the BMoH defined suspected microcephaly cases as newborns with head circumference measures ≤ 33 cm for term (≥ 37 weeks) or < 3^rd^ percentile of the Fenton Preterm Growth Chart for preterm (< 37 weeks) [8] and required mandatory reporting of newborns fulfilling this case definition. This criterion was also applied to cases who were retrospectively identified during this period. From 12 December 2015 to 12 March 2016, the suspected case definition was updated as newborns with head circumference ≤ 32 cm for term and the previous criteria for preterm newborns were maintained [9]. Lastly, on 13 March 2016, the suspected microcephaly case definition was changed to newborns with head circumference < 3^rd^ percentile of the World Health Organization (WHO) Child Growth Standards for term and < 3^rd^ percentile of the INTERGROWTH-21^st^ standards for preterm [10]. Mandatory reporting was performed based solely on head circumference parameters of the newborns, regardless of a Zika diagnosis of the mothers. However, reporting of spontaneous abortions, stillbirths or pregnancies with any detected alterations in the fetal central nervous system in women with a self-reported history of rash during pregnancy was also encouraged regardless of head circumference, but not mandatory [9,10].

### Investigation of reported cases suspected of microcephaly

Salvador, the fourth largest city in Brazil, was one of the north-eastern cities most affected by the microcephaly epidemic [3]. The Salvador Centers for Information and Epidemiologic Surveillance (CIES) is the branch of the Municipal Secretary of Health in charge of the investigation of the reported suspected cases. Investigations were performed by reviewing medical records for intracranial imaging studies. In addition, mothers of reported suspect microcephaly cases were interviewed about clinical manifestations during pregnancy using a standardised questionnaire [10]. After concluding the investigation, CIES updated the national reporting system with the obtained information.

### Image-confirmed congenital brain abnormality case definition

In the present study, we analysed data on suspected microcephaly cases investigated by CIES up to 13 September 2016. According to the availability of data on prenatal or postnatal intracranial imaging studies, the reported cases were classified as either investigated or not investigated. Investigated cases whose intracranial ultrasound, computed tomography, or magnetic resonance imaging results reported intracranial calcifications, ventriculomegaly, dysgenesis or agenesis of the corpus callosum, lissencephaly, cerebellar abnormalities, or anencephaly were classified as confirmed CBA cases. Reports of hydrocephalus or colpocephaly were consolidated as ventriculomegaly. Suspected microcephaly cases that underwent imaging studies and did not exhibit any of the previous findings were excluded from consideration as CBA cases.

### Statistical analysis

An epidemiological curve of the temporal distribution of the suspected microcephaly cases stratified according to the confirmation status was constructed by epidemiological week of the date of birth. Records with incomplete information on date of birth were excluded from the epidemiological curve. Prevalence of CBA per month was calculated dividing the number of imaging confirmed cases by the monthly number of live births from mothers residing in Salvador. To estimate the average annual prevalence of CBA, we divided the CBA prevalence calculated for the complete study period by the number of months in the study period and multiplied the result by 12. Live birth data were obtained at the National Birth Registration System (SINASC) [12].

Clinical characteristics of confirmed and excluded cases of CBA were presented as frequencies and medians. Two-tailed Fisher exact test and odds ratios with 95% confidence intervals (CI) were used to test for differences in the frequencies of clinical characteristics of suspected cases and gestational characteristics of cases’ mothers between confirmed and excluded CBA cases. The Wilcoxon rank test was used to test for difference in maternal age and head circumference between confirmed and excluded CBA cases. In order to investigate whether the imaging-detected CBAs varied according to the presumptive timing of infection during pregnancy, the frequencies of each imaging abnormality in confirmed cases were compared according to the presence and timing of exanthema during pregnancy. A two-tailed significance level of 0.05 was set.

Imaging-confirmed and excluded CBA cases were used to assess the accuracy of different head circumference microcephaly screening criteria for prediction of CBAs. The microcephaly screening criteria evaluated were those adopted by the BMoH from (i) November to 11 December 2015 [8]; (ii) from 12 December 2015 to 12 March 2016 [9]; and (iii) from 13 March 2016 to the present [10]; as well as (iv) the screening criteria recommended by the Pan American Health Organisation (< -2 standard deviation (SD) of the Fenton Preterm Growth Chart according to sex and gestational age for preterm newborns and < 3^rd^ percentile of the WHO Child Growth Standards according to sex for term newborns) [13]; (v) the Fenton Preterm Growth Chart (< -2 SD according to sex and gestational age) [14]; (vi) the INTERGROWTH-21^st^ standards (< -2 SD according to sex and gestational age) [15]; and (vii) the WHO Child Growth Standards for term newborns (< -2 SD according to sex for term newborns) [16]. Some of the criteria require detailed information on gestational age (weeks and days) but gestational age was recorded in full weeks in our dataset. We used the number of weeks plus 0 days in such cases. Accuracy of the criteria in predicting imaging-confirmed CBAs was assessed by calculating sensitivity, specificity, positive and negative predictive values, and their respective 95% CIs. Records with no information on head circumference, gestational age, or with late reporting (> 28 days post birth) without a precise date on which head circumference was measured were excluded from the accuracy analysis. Data were analysed with Stata 14 [17].

### Ethics statement

This investigation was performed using de-identified secondary data obtained by routine activities of the Epidemiological Surveillance Office/Municipal Secretariat of Health from Salvador, Bahia, Brazil. The Salvador Secretariat of Health and the Oswaldo Cruz Foundation Ethics Committee approved the study and granted a waiver of signed informed consent.

## Results

**Figure 1:**
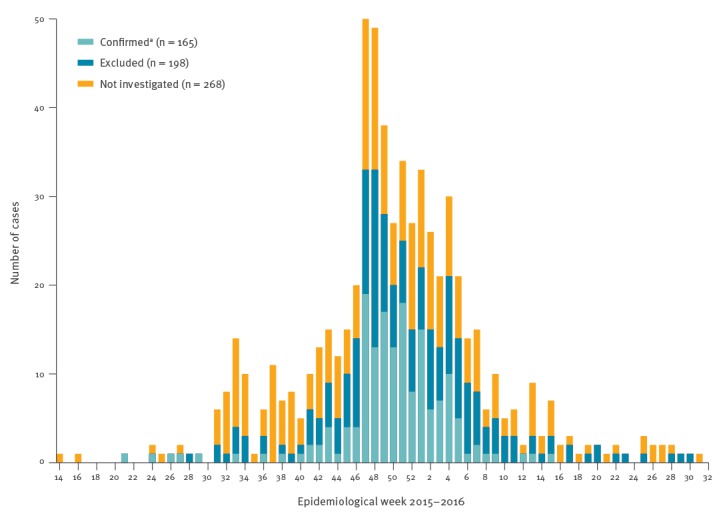
Epidemic curve of the reported cases suspected of microcephaly per week of birth by status of congenital brain abnormality^a^, Salvador, Brazil, April 2015–July 2016 (n = 631^b^)

^a ^Confirmation based on intracranial imaging studies.

^b^ The 631 suspected cases depicted are those with available data on date of birth.

By 13 September 2016, Salvador CIES had received 650 reports of suspected microcephaly cases, who were born between April 2015 and July 2016. Of these, review of medical records to retrieve results of intracranial imaging studies was completed for 365 cases. Among those, 166 (45.5%) had imaging findings consistent with a diagnosis of CBA, while 199 (54.5%) did not. The epidemiological curve of the temporal distribution of reported cases (built for 631 cases with available data on date of birth) peaked between week 47 of 2015 (22–28 November) and week 4 of 2016 (24–30 January) for both the suspected microcephaly reported cases and the imaging-confirmed CBA cases (Figure 1). Prevalence of imaging-confirmed CBA was 1.22 cases per 100 live births in November 2015, 2.24 cases per 100 live births in December 2015, and 1.55 cases per 100 live births in January 2016. Prevalence of imaging-confirmed CBA for the whole study period (April 2015 to July 2016) was 0.34 per 100 live births and the one-year adjusted annual prevalence of image-confirmed CBA was estimated as 0.26 per 100 live births. The last CBA case confirmed by imaging studies during the study period was born in epidemiological week 15 of 2016 (10–16 April).

Male sex was more frequent among the imaging-confirmed CBA cases (45.8%) than among excluded cases (32.7%) (odds ratio (OR): 1.74; 95% CI: 1.11–2.72; p = 0.01) (Table 1). Confirmed cases were more likely to have been born preterm (< 37 weeks) (OR: 6.62; 95% CI: 3.35–13.79; p < 0.001) and had a lower head circumference median (30 cm; interquartile range (IQR): 28–31) compared with excluded cases (32 cm; IQR: 31–32) (p < 0.001). Confirmed cases also had a broader head circumference range (range: 21.5 cm – 42 cm vs 28 cm – 36 cm; p < 0.001). The most frequent CBAs observed among confirmed cases were intracranial calcifications (86.1%) and ventriculomegaly (66.9%) (Table 1). Agenesis of the corpus callosum (12.1%), dysgenesis of the corpus callosum (11.5%), lissencephaly (10.2%), cerebellar abnormalities (5.4%), and anencephaly (1.8%) were identified in a minority of the confirmed cases. Of note, intracranial calcifications were associated with other brain lesions, being present in 83.8% (93/111) of those with ventriculomegaly, but in 19.7% (50/254) of those without it; in 94.1% (16/17) and 36.5% (127/348) of those with and without lissencephaly, in 94.7% (18/19) and 36.1% (125/346) of those with and without dysgenesis of the corpus callosum, and in 85.0% (17/20) and 36.5% (126/345) of those with and without agenesis of the corpus callosum (p < 0.001 for all the comparisons). In terms of other findings, arthrogryposis was found in 6.6% of the confirmed cases, but in none of the excluded cases. Oligohydramnios, intrauterine growth restriction, subependymal cysts, and auditory and ophthalmological abnormalities were also statistically more frequent among the confirmed than the excluded cases (Table 1). Intracranial calcifications were also more frequently observed among those with arthrogryposis than among those without it (72.7% (8/11) vs 38.1% (135/354)) and among those with oligohydramnios than among those without it (66.7% (16/24) vs 37.2% (127/341)) (p < 0.001 for both comparisons).

**Table 1:** Clinical characteristics of suspected microcephaly cases, Salvador, Brazil, April 2015–July 2016 (n = 365)

Clinical characteristics	Congenital brain abnormalities diagnosis			
	Confirmed (n = 166)		Excluded (n = 199)	
	n	%	n	%
Male sex	76	45.8	65	32.7
**Gestational age at birth^a^**				
< 37 weeks	54	33.3	13	7.0
37–42 weeks	108	66.7	172	93.0
**Head circumference**				
Head circumference (cm), median and IQR^b,c^	30.0	28.0–31.0	32.0	31.0–32.0
Head circumference (cm), min–max^b,c^	21.5–42.0		28.0–36.0	
**Intracranial imaging performed**				
Ultrasound	136	81.9	192	96.5
Computed tomography	53	31.9	9	4.5
Magnetic resonance imaging	17	10.2	4	2.0
**Image findings consistent with congenital malformations**				
Intracranial calcifications	143	86.1	0	0.0
Ventriculomegaly	111	66.9	0	0.0
Agenesis of the corpus callosum	20	12.1	0	0.0
Dysgenesis of the corpus callosum	19	11.5	0	0.0
Lissencephaly	17	10.2	0	0.0
Cerebellar abnormalities^d^	9	5.4	0	0.0
Anencephaly	3	1.8	0	0.0
**Other findings**				
Oligohydramnios	17	10.2	7	3.5
Intrauterine growth restriction	14	8.4	2	1.0
Subependymal cysts	16	9.6	7	3.5
Arthrogryposis	11	6.6	0	0.0
Auditory abnormalities^e^	20	19.2	10	11.5
Ophthalmological abnormalities^f^	13	18.8	3	4.5
Death^g^	6	3.6	0	0.0

IQR: interquartile range.

Suspected microcephaly cases may have undergone more than one imaging testing and presented with more than one finding.

^a ^Data available for 162 confirmed cases and 185 excluded cases. The lowest gestational age for the confirmed and excluded cases were 20 and 30 weeks, respectively.

^b ^Data on head circumference were not available for two confirmed cases. Data on head circumference of three confirmed cases and eight excluded cases were not considered in the analysis due to a late measurement without a precise date (> 28 days post birth).

^c ^Some of the cases from both groups of confirmed and excluded congenital brain abnormalities diagnosis had a head circumference size greater than the screening limits used to detect microcephaly. This is because reporting cases of spontaneous abortions, stillbirths or pregnancies with any detected alterations in the fetal central nervous system in women with self-reported history of rash during pregnancy was also encouraged, though not mandatory.

^d ^Cerebellar vermis agenesis or cerebellar hypoplasia.

^e ^Data on auditory abnormalities were available for 104 confirmed cases (86 by auditory screening method, three by brainstem evoked response audiometry test (BERA) and 15 by both) and for 87 excluded cases (79 by auditory screening method, three by BERA test and five by both).

^f ^Data on ophthalmological abnormalities were available for 69 confirmed cases and for 67 excluded cases. Among the confirmed cases, two had pigmentary abnormalities in macula, one abnormal red reflex, one bilateral chorioretinal atrophy and optic disc hypoplasia, one cataracts, one macular atrophy, one corneal excoriation, one chorioretinitis, one optic disc excavation, one coloboma, and three without information on type of abnormality. Among excluded cases, one had retinal haemorrhage, one retinal excavation and one cataracts.

^g ^Death occurred on the day of birth for two cases, within 2 days for one case, within 2 months for two cases and within 4 months for one case.

Maternal age at birth and type of gestation (single vs multifetal) were not associated with imaging confirmation of CBAs (Table 2). Frequency of exanthema during pregnancy among mothers of children in the confirmed group was 73.3%. Among those, 69.4% had the rash during the first trimester and 30.6% during the second or third trimester (22.2% during the second and 8.3% during the third). Mothers of children in the confirmed CBA group were more likely to have had exanthema during pregnancy (OR: 5.04; 95% CI: 3.12–8.19) than mothers of children whose diagnosis of CBA was excluded, especially in the first trimester (OR: 2.91; 95% CI: 1.42–5.97) when compared with the second and third trimester. All other symptoms commonly observed during arboviral infections were more frequent during pregnancy on the mothers of children in the confirmed group (Table 2).

**Table 2:** Maternal clinical characteristics of suspected microcephaly cases, Salvador, Brazil, April 2015–July 2016 (n = 365)

Characteristics	Congenital brain abnormalities diagnosis				OR	95% CI	p value
	Confirmed (n = 166)		Excluded (n = 199)				
	n/N^a^	%	n/N^a^	%			
Mother’s age in years, median and IQR^b^	26	21–32	25	21– 31	NA	NA	0.38
**Type of gestation**							
Multifetal	5/163	3.1	4/195	2.0	1.51	0.32–7.74	0.54
Single	158/163	96.9	191/195	98.0	1	NA	
**Symptoms during pregnancy**							
Fever^c^	66/155	42.3	42/187	22.5	2.56	1.56–4.21	< 0.001
Exanthema	118/161	73.3	68/193	35.2	5.04	3.12–8.19	< 0.001
**Trimester of exanthema^d^**							
First trimester	75/108	69.4	25/57	43.9	2.91	1.42–5.97	0.001
Second or third trimester	33/108	30.6	32/57	56.1	1	NA	
**Other arboviral-infection-like symptoms reported during pregnancy**							
Pruritus	72/133	54.1	36/143	25.2	3.51	2.05–6.04	< 0.001
Arthralgia	60/135	44.4	31/143	21.7	2.89	1.66–5.06	< 0.001
Myalgia	51/135	37.8	25/142	17.6	2.84	1.58–5.17	< 0.001
Headache	48/133	36.1	25/142	17.6	2.64	1.46–4.83	0.001
Retro-orbital pain	24/133	18.1	6/142	4.2	4.99	1.89–15.38	< 0.001
Conjunctival hyperaemia	21/133	15.8	9/142	6.3	2.77	1.16–7.14	0.01

NA: not applicable; CI: confidence interval (95%); IQR: interquartile range; OR: odds ratio.

^a^ The denominators in this column may vary as they are based on the number of cases with known information on the characteristic in question.

^b ^Data on mothers’ ages were not available for nine confirmed and four excluded cases.

^c ^Fever was defined as axillary temperatures of ≥ 37.8 °C.

^d ^Data on trimester of exanthema are presented for the mothers who had exanthema (n = 118 for the mothers of confirmed congenital brain abnormality cases, and n = 68 for those of the excluded cases). For 10 confirmed and 11 excluded cases the trimester of the exanthema was unknown.

When the confirmed cases were classified according to the timing of maternal rash during pregnancy (first, second, third trimester or no rash), there were no statistically significant differences in the frequency of CBAs, nor in the frequency of arthrogryposis, oligohydramnios, and intrauterine growth restriction, between the four groups. An exception was observed for calcifications, that were more frequently present when exanthema occurred during first trimester than when compared with mothers who did not present exanthema during pregnancy (p = 0.02) (Table 3).

**Table 3:** Imaging findings of confirmed congenital brain abnormalities cases, according to the timing of maternal exanthema, Salvador, Brazil, April 2015–July 2016 (n = 151)

Congenital brain abnormalities	Pregnancy trimester of exanthema							
	First (n = 75)		Second (n = 24)		Third (n = 9)		Without exanthema (n = 43)	
	n	%	n	%	n	%	n	%
Intracranial calcifications	68	90.7	23	95.8	7	77.8	32	74.4
Ventriculomegaly	48	64.0	18	75	8	88.9	28	65.1
Agenesis of the corpus callosum	11	14.7	3	12.5	2	22.2	4	9.3
Dysgenesis of the corpus callosum	8	10.7	5	20.8	0	0.0	5	11.6
Lissencephaly	6	8.0	4	16.7	2	22.2	4	9.3
Cerebellar abnormalities^a^	6	8.0	1	4.2	0	0.0	1	2.3
Arthrogryposis	6	8.0	2	8.3	0	0.0	2	4.7
Oligohydramnios	12	16.0	1	4.2	0	0.0	3	7.0
Intrauterine growth restriction	9	12.0	3	12.5	0	0.0	1	2.3
Subependymal cysts	7	9.3	1	4.2	1	11.1	6	14.0

^a ^Cerebellar vermis agenesis or cerebellar hypoplasia.

Among the different head circumference criteria used to screen for microcephaly, the first criterion adopted by the BMoH from November 2015 to December 2015 was the one with the highest sensitivity (83.6%) and lowest specificity (7.3%) in predicting the presence of CBAs (Table 4). On the other hand, the INTERGROWTH-21^st^ standards had the lowest sensitivity (63.4%) and highest specificity (72.4%). Positive predictive value was the highest for the INTERGROWTH-21^st^ standards (63.9%) and the lowest for the criterion adopted by BMoH from December 2015 to March 2016 (41.1%). Negative predictive value was the highest for the WHO Child Growth Standards (77.8%) and the lowest for the BMoH criterion used between November and December 2015 (36.1%).

**Table 4:** Accuracy of different head circumference screening criteria in detecting congenital malformations, Salvador, Brazil, April 2015–July 2016 (n = 365)

Criteria	Sensitivity		Specificity		PPV		NPV	
	%	95% CI	%	95% CI	%	95% CI	%	95% CI
Fenton Preterm Growth Chart	67.7	59.0–75.5	60.6	52.9–67.9	56.6	48.5–64.4	71.1	63.2–78.3
INTERGROWTH-21st standards	63.4	54.7–71.6	72.4	65.1–78.9	63.9	55.1–72.1	72.0	64.7–78.5
WHO Child Growth Standards	75.5	66.2–83.3	54.2	46.3–61.9	51.0	42.9–59.0	77.8	69.2–84.9
Brazilian MoH (Nov–11 Dec 2015)	83.6	76.4–89.3	7.3	3.9–12.1	41.3	35.5–47.3	36.1	20.8–53.8
Brazilian MoH (12 Dec 2015–12 Mar 2016)	79.3	71.6–85.7	11.2	7.0–16.7	41.1	35.2–47.2	40.8	27.0–55.8
Brazilian MoH (13 Mar 2016–present)	68.6	60.2–76.1	55.3	47.7–62.7	54.5	46.9–62.1	69.2	61.0–76.7
Pan American Health Organization recommendation	73.6	65.5–80.7	40.2	33.0–47.8	49.0	42.1–56.0	66.1	56.4–74.9

CI: confidence interval (95%); MoH: Ministry of Health; NPV: negative predictive value; PPV: positive predictive value; WHO: World Health Organization.

## Discussion

In this study, we described a high prevalence of confirmed CBAs in Salvador, as high as 2.2% of the live births in December 2015. The prevalence of image-confirmed CBA estimated for the study period adjusted for one year was 52 times higher than the estimated baseline prevalence of microcephaly in the north-east region (average of 5 cases per 100,000 live births per year, between 2000 and 2014) [18]. Unfortunately, we did not have information on serological or virological ZIKV testing, which would allow ascertaining the aetiology of such an outbreak. However, the peak of births of babies with microcephaly occurred 30–33 weeks after the peak of ZIKV epidemic in Salvador [3], and this is consistent with the growing body of evidence suggesting that the first trimester of pregnancy is the period when ZIKV infections pose the highest risk of adverse fetal outcome [3,19,20]. Taken together, it is reasonable to assume that most of the imaging-confirmed cases in this study were due to congenital ZIKV infection.

As we only considered cases with specific neuroimaging findings as confirmed cases, we certainly underestimated cases of congenital ZIKV infection. Several suspected cases had not been investigated by the time we analysed the data and the imaging modality most commonly used was prenatal or postnatal intracranial ultrasound, which is not an optimal modality to detect abnormalities of the corpus callosum and cerebral cortex. In addition, suspected microcephaly cases were reported based on birth head circumference, which could be well within normal limits in some cases of congenital ZIKV infection [11]. Although reporting of spontaneous abortions, stillbirths and fetuses presenting alterations in the central nervous system was also encouraged, allowing us to confirm a few cases with normal or large head circumference at birth, we could not evaluate whether there was an increase in abortions and stillbirths in Salvador during the study period. On the other hand, some cases counted as confirmed could be due to other causes such as congenital cytomegalovirus infection or genetic disorders, but the number of these cases is expected to be small, considering the baseline rate of microcephaly before the epidemic. In addition, in north-east Brazil (a region in which Salvador was one of the epicenters for the ZIKV outbreak), only 1.3% of confirmed cases of infection-related microcephaly during the 2015–16 period had laboratory evidence of syphilis, toxoplasmosis, cytomegalovirus, or herpes simplex [21]. 

A similar increase in microcephaly cases was reported in other locations where ZIKV epidemics have occurred, such as Colombia, where the prevalence of microcephaly also increased around 6 months after the peak of ZIKV transmission in July 2016. However, the microcephaly prevalence reported in Colombia peaked at 17.7 cases per 10,000 live births, much lower than observed in Salvador [22]. Potential reasons for this difference may include, variable intensity levels of ZIKV transmission, differences in circulating ZIKV strains and different case definitions and surveillance criteria. Further, co-circulation of other arboviruses (dengue and chikungunya, for example), differences in mosquito control measures, and prior exposure to yellow fever vaccination could be contributing factors [22,23]. Additionally, Brazil was the first country in the Americas to experience a large outbreak of ZIKV and to detect an increase in microcephaly cases, and this allowed other countries as Colombia to issue recommendations for delaying pregnancies, which might have resulted in decreased risk of congenital abnormalities associated with ZIKV infection during pregnancy [22].

Female newborns were overrepresented among the reported cases who had a CBA diagnosis excluded. This finding may be due to the application of the same head circumference screening criteria for reporting boys and girls suspected of microcephaly until 12 March 2016 (period during which 85% of the suspect cases had been reported), since head circumference of girls tend to be smaller than boys at the same gestational age [24]. We also found that the frequency of preterm births among the confirmed CBA cases was significantly greater than in excluded cases. Although this finding suggests that congenital Zika syndrome could be associated with preterm birth, we could not determine from the available data whether the early births were natural in their occurrence or due to a medical decision in the presence of fetal anomalies and distress.

The most frequent imaging findings among the confirmed cases were intracranial calcifications and ventriculomegaly. Although these findings are not specific for congenital Zika syndrome, they have been frequently observed among laboratory-confirmed cases of congenital Zika syndrome [25-28]. Anencephaly has not been previously reported among laboratory-confirmed cases of congenital ZIKV infection, and further studies are warranted to determine if it is part of the spectrum of the congenital Zika syndrome. Auditory and ocular manifestations were present in approximately 20% of the confirmed cases. However, they were also found in lower frequencies among the suspected microcephaly cases with normal intracranial imaging studies. Since these manifestations have been linked to congenital ZIKV infection [29,30], it is important to further investigate whether ZIKV infection can cause auditory or ocular lesions in the absence of structural malformations in the brain and to monitor for long-term consequences in ZIKV-exposed babies born with no alterations in brain imaging studies. In addition, as preterm babies may have more auditory and visual complications than term babies, we investigated whether these sensory disorders were associated with premature delivery and found a higher frequency of auditory abnormalities among preterm than term babies (26.5% vs 11.4%; p = 0.01), but this difference was not observed for ophthalmological abnormalities. Further studies are necessary to determine the specific contribution of both prematurity and ZIKV-related neurological injury on the occurrence of sensory disorders in these children.

Frequency of exanthema during pregnancy among mothers of children in the confirmed group was 73.3%. It has been previously estimated that only 20% of the ZIKV infections are symptomatic [31], but other studies have shown similarly high frequencies of exanthema among mothers who gave birth to children with congenital Zika syndrome [7,32,33]. Recall bias and the surveillance system associating a rash during pregnancy as a marker for microcephaly risk may have accounted for the high proportion of symptomatic women in this series. However, both confirmed and excluded CBA cases originated from the same reported dataset and only 35.2% of the mothers of children in the excluded group reported a rash, representing a significant difference. Therefore, it is likely that symptomatic ZIKV infection during pregnancy truly poses a higher risk of CBAs. Similar findings of an association of rash during pregnancy with increased CBA risk was previously reported in Brazil [7,32,33], although an absence of such association was noted in the United States (US) [34].

Among mothers of children in the confirmed group who reported a history of rash during pregnancy, 69.4% had the rash during the first trimester, but, in addition, 22.2% had rash during the second trimester and 8.3% during the third trimester. Data linking ZIKV infection in the second and third trimester to congenital malformations are still scarce [27,32], and our findings reinforce that second and third trimester infections may also lead to congenital Zika syndrome with CBAs.

Head circumference-based criteria were primarily used during the epidemic of congenital ZIKV infection. These can be easily applied in any clinical setting and do not require any special equipment. Although microcephaly in general is a risk factor for developmental delay, according to the US National Collaborative Perinatal Project, which followed a cohort of newborns, only 11% of children with microcephaly (head circumference ≤ -2SD) at birth had IQ ≤ 70 at 7 years of age [35], suggesting that microcephaly has a relatively low specificity in predicting poor neurodevelopmental outcome in the general population. Further, cases with congenital ZIKV infection without microcephaly at birth have been reported [36]. On the other hand, the severity of anomalies in neuroimaging during the neonatal period has been shown to have a good prognostic value for predicting poor developmental outcomes in symptomatic congenital cytomegalovirus infection [37,38], which shares many clinical and radiological similarities to congenital ZIKV infection. Therefore, it is important to evaluate the accuracy of different microcephaly criteria in predicting CBAs. 

In this regard, none of the criteria performed particularly well. Overall, the INTERGROWTH-21^st^ standards had a better performance, with sensitivity, specificity and positive and negative predictive values over 60%. A significantly low specificity of the criteria used by the BMoH until 11 March 2016 was also noted. There is only one small prior study with 31 cases that used imaging findings consistent with congenital infection as the reference criteria for sensitivity estimation of different head circumference criteria [39]. Compared with this prior study, our study revealed lower sensitivity and much lower specificity. Combined with relatively low positive and negative predictive values, these data clearly demonstrate the limitations of head circumference in accurately identifying children with CBA during ZIKV epidemics.

Novel screening methods for congenital Zika syndrome during ZIKV epidemics that incorporate additional parameters to head circumference are urgently needed in order to detect the maximum number of affected children and fetuses for further specific health assistance, without compromising specificity [40,41]. Prenatal and postnatal intracranial ultrasound triage performed by experienced ultrasonographers may be an efficient approach during outbreaks, especially for women who experienced symptoms consistent with ZIKV infection during pregnancy. However, the importance of clinical examination and follow-up of newborns, as well as development of better serological and molecular tests, cannot be understated.

Our study highlights the magnitude of neurological consequences of the ZIKV epidemic in Salvador, Brazil, further delineates congenital Zika syndrome, and identifies limitations of screening for congenital Zika syndrome based on head circumference that was performed during the recent epidemics. Follow-up studies of children with and without microcephaly or congenital abnormalities, who were exposed to ZIKV in utero are needed to fully understand the full spectrum of congenital Zika syndrome.
